# Supplementary material for: Geometry and dynamics of representations in a precisely balanced memory network related to olfactory cortex
Source: eLife. 2025 Jan 13;13:RP96303. doi: 10.7554/eLife.96303 (PMC11733691; doi:10.7554/eLife.96303)
Supplement: MDAR checklist [file elife-96303-mdarchecklist1.docx]

**MDAR Checklist**

**Materials**

N/A

**Design**

| **Statistics** | **Indicate where provided:**  **Section/legend** | **N/A** |
| --- | --- | --- |
| Describe statistical tests used and justify choice of tests. | Information can be found in Figure 5 legend |  |

**Analysis**

| **Code availability** | **Indicate where provided:**  **section/figure legend** | **N/A** |
| --- | --- | --- |
| For any computer code/software/mathematical algorithms essential for  replicating the main findings of the study, whether newly generated or  re-used, the manuscript includes a data availability statement that  provides details for access or notes restrictions. | Information can be found in the Key Resource Table and in the “DATA AND CODE AVAILABILITY” section |  |
| Where newly generated code is publicly available, provide accession  number in repository, OR DOI OR URL and licensing details where  available. State any restrictions on code availability or accessibility. | Information can be found in the “DATA AND CODE AVAILABILITY” section |  |
| If reused code is publicly available provide accession number in  repository OR DOI OR URL, OR citation. |  | N/A |

**Reporting**

N/A
